# Mitochondrial genome of *Tesseropora rosea*: molecular evidence for non-monophyly of the genus *Tetraclita*

**DOI:** 10.1080/23802359.2017.1422412

**Published:** 2018-01-05

**Authors:** Yue Feng Cai, Xin Shen, Liang Zhou, Ka Hou Chu, Benny Kwok Kan Chan

**Affiliations:** a Jiangsu Key Laboratory of Marine Biotechnology, Jiangsu Institute of Marine Resources, Huaihai Institute of Technology, Lianyungang, China;; b Co-Innovation Center of Jiangsu Marine Bio-industry Technology, Lianyungang, China;; c Simon F. S. Li Marine Science Laboratory, School of Life Sciences, The Chinese University of Hong Kong, Hong Kong, China;; d Biodiversity Research Center, Academia Sinica, Taipei, Taiwan

**Keywords:** *Tetraclita*, *Tesseropora rosea*, mitochondrial genome, phylogeny, non-monophyly

## Abstract

The complete mitochondrial genome of *Tesseropora rosea* (Tetraclitidae) was presented. The genome is a circular molecule of 15,330 bp, which encodes 13 PCGs, two rRNA genes, and 22 tRNA genes. The length of all non-coding regions is 768 bp, with the longest one speculated as the control region (255 bp), which is located between *12S rRNA* and *trnK*. Phylogenetic analysis based on mitochondrial PCGs shows that *T. rosea* is nested within the genus *Tetraclita*, more closely related to *Tetraclita japonica* than to *Tetraclita rufotincta* or *Tetraclita serrata*. Thus, the monophyly of the genus *Tetraclita* is not supported.

Barnacles (Crustacea: Cirripedia) are sessile, filter-feeding crustaceans that occur from the intertidal to the deep sea, in almost all marine and estuarine habitats (Pasternak and Achituv 2007; Chan et al. 2009). *Tesseropora* is found from northern Queensland to eastern Victoria, Australia (Endean et al. 1956) and is most common on rocky shores exposed to strong wave action (Underwood 1981). *Tesseropora rosea* is the most abundant sessile animal at mid-tidal level on shores exposed to wave action (Fairweather 1988). In this study, we characterized the complete mitochondrial genome sequence of *T. rosea*, which would contribute to further physiological, molecular, and phylogenetical studies of this barnacle.

Specimen of *T. rosea* was collected from Sydney, Australia, which was deposited in the Biodiversity Research Museum, Academia Sinica, Taiwan. The muscle tissue isolated from the fresh specimen was immediately preserved in 95% ethanol. Total DNA was extracted from the muscle tissue with the QIAamp Tissue Kit (QIAGEN, Hilden, Germany) following the manufacturer’s recommendations. The amplifications of internal fragments and long fragments followed the procedures described in our previous study (Shen, Tsang, et al. 2015). Sanger sequencing was conducted by an ABI 3730XL analyzer (Applied Biosystems, Foster City, CA).

The mitochondrial genome of *T. rosea*, with a total length of 15,330 bp, encodes 13 protein-coding genes (PCGs), two rRNA genes, and 22 tRNA genes (GenBank Accession no.: KY865099). The contents of A, G, T, and C are 34.92%, 11.93%, 33.21%, and 19.94%, respectively. The length of coding sequences is 11,043 bp (72.04%), which was the lowest among the available mitochondrial genomes of Tetraclitidae. The total length of non-coding regions is 768 bp, with the longest one as the putative control region (255 bp), which is located between *12S rRNA* and *trnK*. Twelve PCGs in *T. rosea* start with ATR (ATG or ATA), while *cox1* starts with TTG. Remarkably, *cox3*, *nd3*, and *nd4* ended by T–– as the stop codon and the remaining PCGs have the complete stop codon TAA or TAG. The A + T contents of *12S rRNA* and *16S rRNA* are 67.00% and 73.56%, respectively.

To elucidate phylogenetic relationships of *T. rosea* with the other barnacles, we used MEGA6.0 (Tamura et al. 2013) to construct a maximum likelihood tree based on 13 PCGs from 20 complete mitochondrial genomes of members from the infraclass Cirripedia (Shen, Chan, et al. 2015; Shen, Tsang, et al. 2015; Tsang, Shen, et al. 2015; Wares 2015; Baek et al. 2016; Shen, Chan, et al. 2016; Shen, Chu, et al. 2016; Shen, Tsoi, et al. 2016; Shen et al. 2017). Within the order Tetraclitidae, *T. rosea* was found to cluster with *Tetraclita japonica* (BP = 98), and then other two *Tetraclita* species, *Tetraclita rufotincta* (BP = 83) and *Tetraclita serrata* (BP = 100), and was more closely related to *T. rufotincta* than to *T. serrata* (Figure 1). This result is consistent to the topology of the three species based on a phylogenetic analysis using seven genes (mitochondrial COI, 16S, and 12S rRNA, and nuclear EF1a, RPII, H3, and 18S rRNA) (Tsang, Chu, et al. 2015), thus providing further support that the genus *Tetraclita* does not constitute a monophyletic assemblage. In conclusion, the complete mitochondrial DNA of *T. rosea* is decoded for the first time in this study, providing data for further molecular and evolutionary analysis of barnacles.

**Figure 1.**
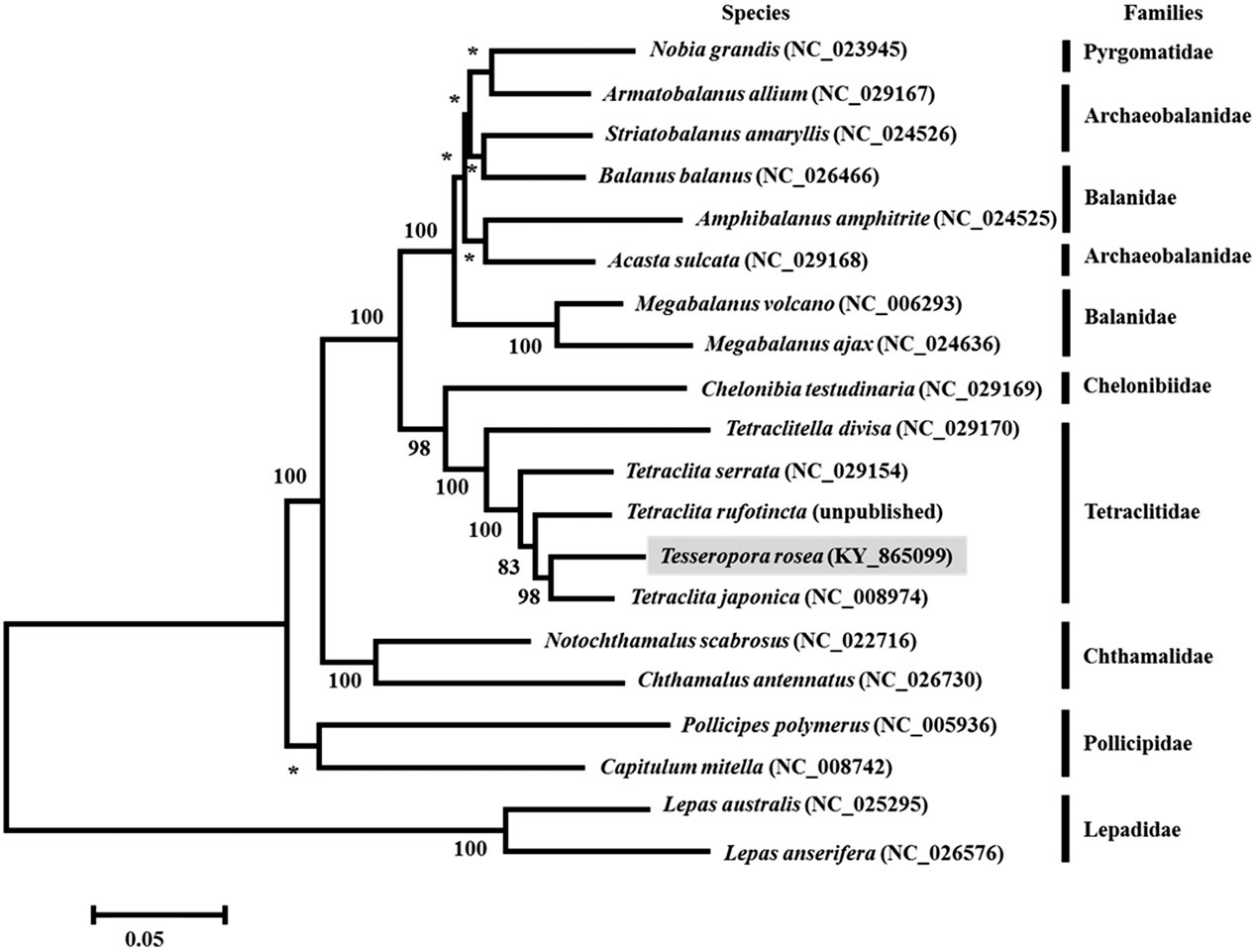
Maximum-likelihood phylogenetic tree of amino acid sequences in 13 PCGs of *Tesseropora rosea* and 19 other Cirripedia species. Nodal supports are denoted on the corresponding branches for a bootstrap (BP) value >50%, while asterisks indicate BP ≤50%.
